# Phylogenetic Group of* Escherichia coli* Isolates from Broilers in Brazilian Poultry Slaughterhouse

**DOI:** 10.1155/2017/5898701

**Published:** 2017-10-10

**Authors:** Fernanda M. Coura, Soraia A. Diniz, Marcos X. Silva, Thiago L. M. Arcebismo, Silvia Minharro, Adriana C. F. Feitosa, Andrey P. Lage, Terezinha Knöbl, Jamili Maria Suhet Mussi, Marcos B. Heinemann

**Affiliations:** ^1^Escola de Veterinária, Departamento de Medicina Veterinária Preventiva, Universidade Federal de Minas Gerais (UFMG), Av. Antônio Carlos, No. 6627, 30123-970 Belo Horizonte, MG, Brazil; ^2^Escola de Medicina Veterinária e Zootecnia, Inspeção e Tecnologia de Carnes e Derivados, Universidade Federal do Tocantins (UFT), BR-153, Km 112, 77804-970 Araguaína, TO, Brazil; ^3^Serviço de Inspeção Federal, Ministério da Agricultura, Pecuária e Abastecimento, SISA, Palmas, TO, Brazil; ^4^Departamento de Patologia, Faculdade de Medicina Veterinária e Zootecnia, Universidade de São Paulo, Av. Prof. Orlando Marques de Paiva, No. 87, 05508-270 São Paulo, SP, Brazil; ^5^Departamento de Medicina Veterinária Preventiva e Saúde Animal, Faculdade de Medicina Veterinária e Zootecnia, Universidade de São Paulo, Av. Prof. Orlando Marques de Paiva, No. 87, 05508-270 São Paulo, SP, Brazil

## Abstract

The aim of the study was to determine the phylogenetic groups of* E. coli *strains isolated from seemingly healthy broiler and broiler condemned suspected of colibacillosis in a Brazilian slaughterhouse. Samples from respiratory tract and edible giblets (liver and heart) of broilers with and without macroscopic lesions of colibacillosis were collected at slaughter. There were 84 strains isolated from broilers condemned of which 11 were obtained from swabs of the heart, 7 from the liver, and 66 from the respiratory tract. Of the 53* E. coli *strains isolated from broilers not condemned, 5 were isolated from the heart, 4 from the liver, and 44 from the respiratory tract.* E coli *strains were tested via PCR for phylogenetic groups A, B1, B2, C, D, E, and F. Phylogroups A and B1 were the most common phylogroups of* E. coli *obtained from healthy and sick-appearing broiler carcasses. The results of the study showed that phylogroups B2 and E were associated with the heart samples and phylogroup A was associated with respiratory tract samples, phylogroup B1 with not condemned carcass, and phylogroup D with liver samples.

## 1. Introduction

Brazil is the second largest producer of chicken meat and the largest exporter of this product [1]. Moreover, the Brazilian poultry industry is expanding to other regions beside southern Brazil (the traditional hub of poultry production) to include the Central/Western region of the country [2]. Currently, one of the greatest challenges for Brazilian poultry producers is to reduce the economic losses caused by the condemnation of carcasses in slaughterhouses, including the air sac disease, due the* E. coli* infection [3].

*E. coli *is a member of the normal microbiota in poultry intestine but the colonization of the respiratory tract by pathogenic* E. coli *strains is associated with extraintestinal disease, resulting in morbidity and mortality of poultry by septicemia [4, 5].

The pathogenesis of colibacillosis is not completely understood, but some* E. coli* have many virulence properties associated with host tissue colonization, iron uptake systems, serum resistance, production of toxins, and defensins [5]. These strains were classified as Avian Pathogenic* E. coli* (APEC), a subpathotype of the Extraintestinal Pathogenic* E. coli* (ExPEC).

The molecular detection of virulence genes by PCR is the most common approach for the study of ExPEC. However, there is no consensus about the minimum predictive factors of APEC, since this subset group of strains is quite heterogenous [6–8]. In addition, while APEC acts as a primary pathogen, some commensal strains of* E. coli*, devoid of virulence factors, can also cause colisepticemia, acting as opportunistic secondary pathogens in stressed and immunocompromised birds, mainly in the presence of respiratory diseases as infectious bronchitis or mycoplasmosis [3, 5].

*E. coli *consists of commensal and pathogenic strains of great diversity, and the classification of these microorganisms in phylogenetic groups through the combination of gene clusters is important to understand* E. coli *pathogenesis and interaction with its hosts [9]. An understanding of the* E. coli *genomic structure showed that the strains belonging to the different phylogroups may be associated with the source of isolation [10]. Since its introduction in 2000 [11], phylogenetic typing using PCR has become widely used due to its simplicity and speed, allowing the differentiation of virulent strains (B2 or D) and commensal lineages (A and B1). This method was improved in 2013 [10] and a quadruplex PCR can identify seven phylogroups (A, B1, B2, C, D, E, and F).

Phylogenetic characterization is an important tool to improve the understanding of* E. coli *population and the relationship between strains and disease [12]. Due to the diversity and importance of* E. coli* infections in poultry as well as the economic impact of these infections, this study determined the phylogenetic groups of* E. coli *isolated from healthy-appearing broiler carcasses and carcasses condemned as suspected of colibacillosis at slaughter. 

## 2. Materials and Methods

### 2.1. Sample Collection and Bacterial Examination

Samples from respiratory tract and edible giblets were collected from one slaughterhouse under Federal Sanitary Inspection localized in Tocantins State, Brazil, from May 2011 to April 2012. This facility slaughters broiler chickens from São Paulo, Tocantins, Goiás States, and the Federal District. Lesions were classified during slaughter by the Federal Inspectors of Brazilian Ministry of Agriculture, and slaughter followed the Brazilian Ministry of Agriculture guidelines [13].

Samples from the respiratory tract (trachea and air sacs) and the edible giblets liver and heart of broilers without macroscopic lesions (which were not condemned and passed the inspection) were collected after chilling process. Swabs from the trachea, air sacs, liver, and heart from carcasses condemned as suspected of colibacillosis were also collected during inspection of the carcass and the corresponding viscera at the evisceration line. The swabs of each organ were placed in a sterile test tube containing 0.9 mL of 0.85% saline and refrigerated until processing. The samples were appropriately packed in an isothermal box with ice packs and transported to the School of Veterinary and Animal Science at the Federal University of Tocantins for bacteriological culture of* E. coli*.

The swabs were processed individually according to [14] for isolation and identification of* E. coli.* Briefly, swabs were streaked onto MacConkey Agar and incubated at 37°C for 24 h. Then, after checking the growth of colonies on MacConkey Agar, up to three lactose positive and negative colonies were characterized biochemically according to [14] using agar triple sugar iron (TSI), citrate, indole, methyl red, and urea tests. Tubes were then incubated at 37°C for 24 h. Only one confirmed lactose positive strain was chosen from each carcass sample for further analysis.* E. coli *strains were maintained in Nutrient Agar at room temperature until molecular analysis.

*E. coli *strains were obtained from 59 batches in the abattoir. The distribution of batches per state is shown in Table 1. In each batch of animals to be slaughtered, 10 samples were collected, 5 from healthy-appearing broiler carcasses and 5 from carcasses condemned by colibacillosis at slaughter.

### 2.2. DNA Extraction and Phylogenetic Group Determination

*E. coli* strains were sent to the Laboratory of Applied Bacteriology at the Veterinary School of The Federal University of Minas Gerais for phylogenetic group determination. Bacterial DNA was obtained by plating previously isolated colonies of* E. coli* onto MacConkey Agar. After plating, the samples were incubated for 18–24 h at 37°C. The bacterial DNA was then obtained by resuspending the subcultured cells in 1.5 mL microtubes containing 100 *µ*L of TE (10 mM Tris-HCl; EDTA 1 mM, pH 8.0); DNA was extracted according to [15].* E. coli *strains were assigned to one of the seven phylogenetic groups A, B1, B2, C, D, E, and F based on the presence and absence of the gene or DNA fragments* chuA, yjaA*,* arpA, and trpA* genes and TSPE4.C2 via PCR assay according to [10]. The typing scheme to classify* E. coli *in the phylogroups is well described in the article of [10] and was performed in the present study accordingly. The EDL933 (phylogroup E) and E2348/69 (phylogroup B2) were used as positive controls.

### 2.3. Statistical Analysis

The chi-squared test was used at 95% significance to estimate the differences among the proportion of phylogenetic groups identified in the organs of broilers and to assess the proportion of phylogenetic groups in colisepticemic and healthy carcasses.

Correspondence analysis (CA) [16] was used to study the phylogroups, origin of the samples (liver, heart, and respiratory tract), and the characteristic of the carcass/viscera at inspection (condemned or not condemned) using the Stata®/SE 12.0 [17]. In CA analysis, the relationship between the categories was represented in a two-dimensional graph with the value of the third dimension shown in parentheses. The degree of relation between the categories was demonstrated by evaluating which variables were plotted closely together. The CA examines the relationship between categorical and nominal data using a contingency table of the categorical variables and transforms nonmetric data to a metric data, allowing data to be mapped and visualized [18].

**Figure 1:**
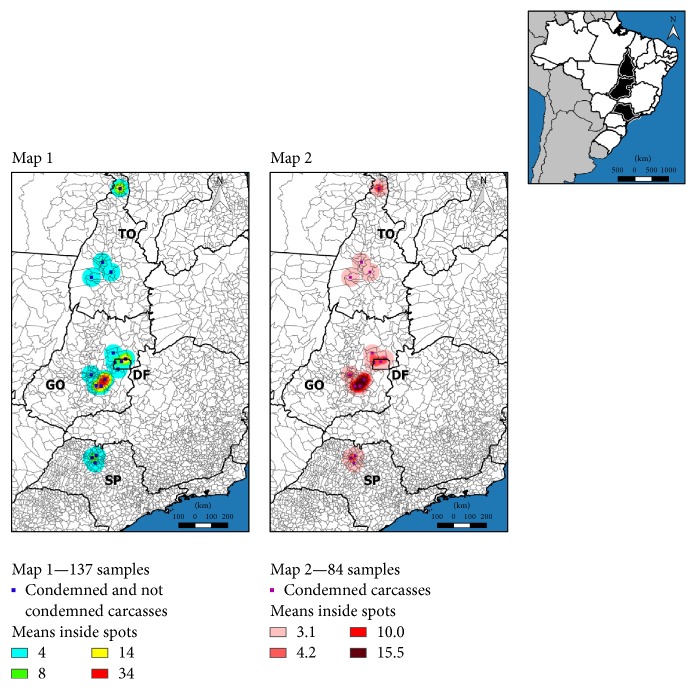
Spatial kernel density estimator of the total* E. coli *strains isolated in the respiratory tract, liver, and heart of broilers at a slaughterhouse according to the city of origin of the batch of broilers (Map 1) and the kernel density estimator of the* E. coli *strains isolated from the respiratory tract, liver, and heart of broilers condemned suspect of colibacillosis according to the origin of the batch of broilers (Map 2).

**Figure 2:**
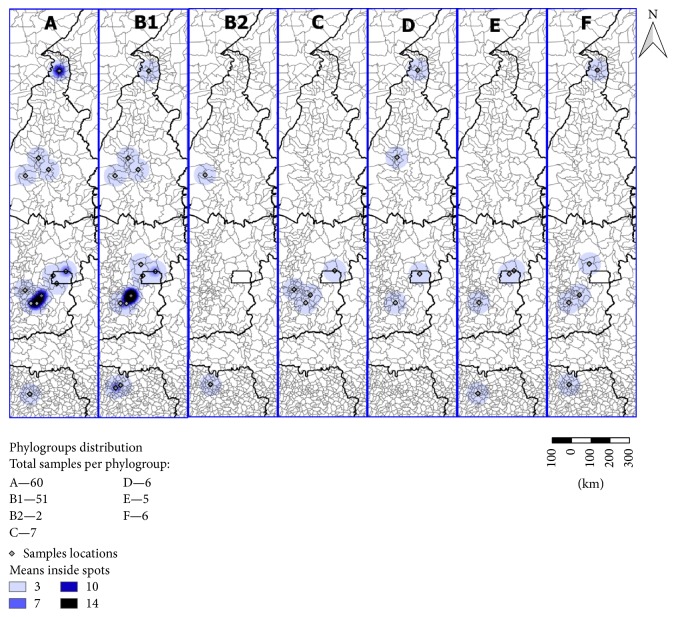
Spatial kernel density estimator of the distribution of the phylogenetic groups of* E. coli *strains isolated from the respiratory tract, liver, and heart of broilers at a slaughterhouse according to the city of origin of the batch of broilers to be slaughtered.

All spatial analytical procedures were performed with program Quantum GIS [19]. Two maps were created to get the georeference of the* E. coli *strains isolated from the samples collected (Figure 1). One was for all strains of* E. coli *isolated from the respiratory tract and the edible giblets (Figure 1/Map 1) and the other was for* E. coli *strains isolated from samples collected from condemned broilers (Figure 1/Map 2).  Figure 2 was created to show the distribution of the phylogenetic groups of* E. coli *strains isolated from broilers at slaughter (condemned and not condemned) according to the origin of the batch of animals to be slaughtered. The coordinates of the samples were obtained by taking the centroid point of the municipality to which they belonged—this created a layer of points. These points were submitted to an adaptive radius with a quartic density kernel to illustrate the concentration of the samples as many of them belonged to the same location.

## 3. Results

In total, 59 batches of animals were analyzed, a total of 590 broiler carcasses. One hundred and thirty-seven strains of* E. coli *were isolated and used in the study.* E. coli *isolates were obtained from respiratory tract (trachea and air sacs) and edible giblets (liver and heart) of broiler carcasses. There were 84 strains isolated from broilers condemned suspected of colibacillosis of which 11 were obtained from swabs of the heart, 7 from the liver, and 66 from the respiratory tract. Of the 53* E. coli *isolated from samples of inspection passed broilers, 5 were isolated from the heart, 4 from the liver, and 44 from the respiratory tract.  Table 2 shows the phylogenetic group distribution of* E. coli *obtained from samples of condemned and not condemned broilers slaughtered in a Brazilian slaughterhouse. The phylogenetic groups of* E. coli *according to the origin of isolation in the carcasses are shown in Table 3. There was no association between phylogroups and the condition of the carcasses (*P* = 0.20), which means condemned or not condemned. Phylogroups B2 (*P* < 0.001) and E  (*P* = 0.044) were associated with the heart samples.

**Figure 3:**
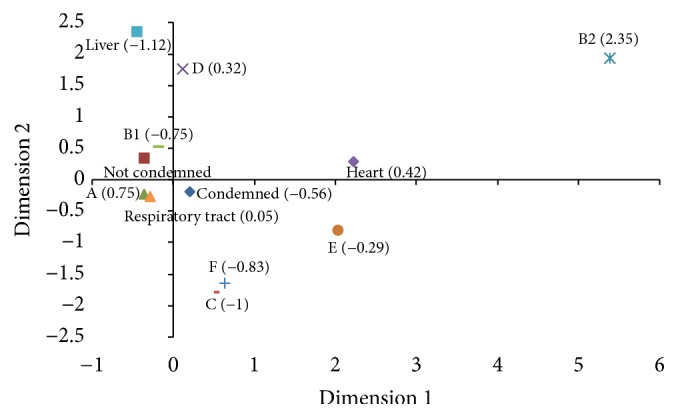
Correspondence analysis for the categories. This two-dimensional representation explains 42.52% of the total variation with 15.59% explained by 1st dimension, 13.86% by the 2nd dimension, and 13.07% by the 3rd. The value of the third dimension is shown in parenthesis.

We used CA to analyze the degree of relationship between the categories phylogroups, origin of the sample (liver, heart, and respiratory tract), and characteristic of the carcass at the inspection (condemned or not condemned) (Figure 3). The two-dimensional representation explains 42.52% of the total variation with 15.59% explained by the 1st dimension, 13.86% by the 2nd dimension, and 13.07% by the 3rd. According to this analysis, phylogroup A was plotted near the respiratory tract samples, while phylogroup B1 was near the not condemned respiratory samples. Samples obtained from condemned broilers were plotted close to both phylogroups A and B1; the liver samples were close to phylogroup D. Although phylogroups B2 and E were plotted away from the other variables, both phylogroups were closer to the heart samples than the other variables.

Figure 1 shows the kernel function of the total* E. coli *strains isolated in the slaughterhouse according to the city of origin of the broiler chickens' batches to be slaughtered (Figure 1/Map 1) and the kernel function of the* E. coli *strains isolated from broiler chickens condemned suspect of colibacillosis according to the origin of the batch of the broilers (Figure 1/Map 2). The map showed that* E. coli *strains isolated from healthy and sick-appearing broiler carcasses originated mainly from batches of animals from the Goiás State and the Federal District.  Figure 2 shows the distribution of the phylogenetic groups of* E. coli *strains isolated from the respiratory tract and the edible giblets of broiler chickens at slaughter according to the origin of the batch of broilers to be slaughtered and showed that phylogroups A and B1 were detected in São Paulo, Tocantins, Goiás States and the Federal District, but mostly in Goiás State; phylogroup B2 in São Paulo and Tocantins States; phylogroup C in Goiás State; phylogroup D in Tocantins and Goiás States and the Federal District; phylogroup E in São Paulo and Goiás States and the Federal District; phylogroup F São Paulo, Tocantins and Goiás States.

## 4. Discussion

A PCR technique to identify the seven phylogenetic groups was recently developed by Clermont et al. [10], and its use is rare in the literature [20–22]. The cited articles in the discussion section of the present study used the triplex PCR-based method, which identifies the phylogroups A, B1, B2, and D. Previously, our group showed the relation of the seven phylogroups according to the host (cattle calves, buffalo calves, and poultry) [20]. Therefore, to the best of our knowledge, this is the first report that analyzed the association of the seven phylogroups of* E. coli*, the origin of the samples (liver, heart, and respiratory tract), and the characteristic of the carcass/viscera at inspection (condemned or not condemned) using the quadruplex PCR-based method in Brazil.

Our study showed that phylogroups A followed by B1 are the most common phylogroups of* E. coli *obtained from not condemned broiler carcasses. This suggests that systemic contamination occurs via the respiratory tract because* E. coli* strains from the respiratory tract are primarily phylogroup A and to a lesser extent B1. The main form of infection with* E. coli* is via inhalation of feces-contaminated dust [23]. Koga et al. [24] suggest that poultry carcass contamination occurs by commensal* E. coli*. This observation is reinforced by the fact that* E. coli *isolated from the healthy group of our study was mainly phylogroup A. Moreover, the CA showed a relationship between phylogroup A strains and the respiratory tract—an organ considered to be the first step of systemic infection by* E. coli *[5]. In addition, commensal strains of* E. coli* may act as opportunistic agents in secondary infections in lots of birds affected by infectious bronchitis virus and other endemic respiratory pathogens in Brazil.

On the contrary, phylogroups B1 followed by A are the most common in strains obtained from condemned broilers. Similar results were found in Brazil [25, 26], Japan [27], Iran [28], and Australia [29]. A study in South Brazil showed that the percentage of phylogroup B1 isolated in refrigerated chicken carcasses increased over time while phylogroups B2 and D decreased [24]. Moreover, phylogroups A and B1 strains predominate in birds [20, 30], similar to our findings. However, phylogroups A and D were the most common phylogroups of* E. coli *isolated from poultry in Italy [31], China [32], Canada [33], and Iran [34], indicating that the frequency of phylogroups might vary between different geographic regions.

Overall, our results show that phylogroups B2, C, D, E, and F are not common* E. coli* phylogroups isolated from broilers. These results agree with other studies [20, 24, 31, 32]. On the contrary, phylogroup B2 was detected at high frequencies in other studies [25, 26, 35]. Phylogroup D was also detected at a low frequency (4.37%). In the CA, phylogroup D fell close to the liver samples in the CA map, while phylogroups B2 and E fell close to and associated with heart samples. In a Brazilian study,* E. coli *isolated from cellulitis lesions in broiler chickens were predominated by phylogroup D [36]. Phylogroups B2 and D strains are considered extraintestinal pathogenic* E. coli *[37] while phylogroup E includes human and animal intestinal strains and human EHEC O157:H7 strains [38]. In sum, our results suggest that phylogroups B2 and D are also extraintestinal pathogenic* E. coli *for broilers and indicate that phylogroup E may be associated with extraintestinal pathogenic* E. coli *for broilers, but further studies are needed to confirm this association, since the number of phylogroup E strains identified in the study was low.

The kernel function showed that the* E. coli *strains isolated originated mainly from batches from the Goiás State and the Federal District. The number of broilers slaughtered under Federal Inspection in the Middle-West region of Brazil has increased over the years, and the main cause of condemnation is colibacillosis and airsacculitis. In addition,* E. coli* strains were the main bacterial agent isolated in slaughterhouses with Federal Inspection in the State of Goiás [39]. Over 90% of Brazilian poultry production is based on integrated systems with good sanitary status, quality, and sustainability. The slaughterhouses are under the Federal Inspection Service to minimize risk. Colibacillosis in poultry is not well understood and is highly associated with the presence of risk factors such as stress, presence of other pathogens, and immunity status of birds [5].

Studies suggest that APEC is a reservoir of human ExPEC and should be considered a potential zoonotic risk [35, 40]. Our study did not evaluate the presence of virulence genes, but ExPEC phylogroups B2 and D were detected. Studies on the phylogenetic background of* E. coli *are important because there are data showing a link between phylogeny and virulence. Here, the background is important for the acquisition and expression of different virulence factors and epidemiology studies [41]. Over 95% of* E. coli *strains can be correctly assigned using the extended quadruplex method [10].

## 5. Conclusions

Our results indicate that all phylogroups are detected in* E. coli *isolated from healthy and sick-appearing broiler carcasses slaughtered in Brazil, but phylogroups A and B1 are the most common phylogroups. Phylogroup A was isolated mainly from the respiratory tract, while B1 was mainly detected in carcasses condemned. Phylogroups B2 and E were associated with* E. coli *isolated from heart samples, while phylogroup D was associated with liver samples.

## Figures and Tables

**Table 1:** Distribution of batches of animals to be slaughtered according to origin of sample to be collected and the state of the batches.

Samples	GO	TO	DF	SP	Total
Respiratory tract	17	6	11	1	35
Heart	4	4	2	3	13
Liver	5	1	3	2	1
Total	26	11	16	6	59

**Table 2:** Distribution of phylogroups of *E. coli *strains isolated from respiratory tract, liver, and heart of broilers condemned and not condemned during inspection.

Condition of the carcasses	Phylogroup							
	A	B1	B2	C	D	E	F	Total
Condemned	30	35	1	6	3	4	5	84
Not condemned	30	16	1	1	3	1	1	53
Total	60	51	2	7	6	5	6	137

**Table 3:** Distribution of phylogroups of *E. coli *strains isolated from the heart, liver, and respiratory tract of broiler during inspection.

Organ of origin	Phylogroup							
	A	B1	B2	C	D	E	F	Total
Heart	5	4	2^*∗*^	1	1	2^*∗*^	1	16
Liver	3	7	0	0	1	0	0	11
Respiratory tract	52	40	0	6	4	3	5	110
Total	60	51	2	7	6	5	6	137

^*∗*^Identification of phylogroups B2 (*P* < 0,001) and E  (*P* = 0,044) was associated with heart samples.
